# Assessment of Different Niobium-Based Catalytic Systems for 5‑Hydroxymethylfurfural Production from Glucose and Microcrystalline Cellulose

**DOI:** 10.1021/acsomega.5c12543

**Published:** 2026-04-14

**Authors:** Jéssica S. M. M. T. Nogueira, Livia M. Carneiro, João P. A. Silva, Solange I. Mussatto, Inês C. Roberto

**Affiliations:** † Department of Biotechnology, Engineering School of Lorena, University of São Paulo, Estrada Municipal do Campinho, 100, Lorena, São Paulo 12602-810, Brazil; ‡ Department of Chemical Engineering, Engineering School of Lorena, University of São Paulo, Estrada Municipal do Campinho, 100, Lorena, São Paulo 12602-810, Brazil; § Department of Biotechnology and Biomedicine, Technical University of Denmark, Søltofts Plads, Building 223, 2800 Kongens Lyngby, Denmark

## Abstract

This study investigates the production of 5-hydroxymethylfurfural (HMF) from glucose and microcrystalline cellulose using niobium-based catalytic systems in acetone/water media, under short reaction times. Three catalytic strategies—individual, hybrid, and impregnated—were evaluated by combining niobium pentoxide (Nb_2_O_5_) or niobium phosphate (NbOPO_4_) with phosphotungstic acid (HPW), in both powder and pellet forms. Among these systems, NbOPO_4_ powder exhibited the best performance for glucose conversion, achieving a yield of 38.5% and a volumetric rate of 26.9 g/L·h, while cellulose conversion required an HPW/NbOPO_4_ impregnated system, yielding 18.9% with a volumetric productivity of 88.1 g/L·h. Further, recyclability tests for glucose conversion showed that NbOPO_4_ powder remained active for seven consecutive cycles, whereas the pellet formed partially deactivated after four cycles. Together with the use of relatively green solvents, short reaction times, and noncorrosive conditions, these results support the development of efficient, reusable, and environmentally friendly catalytic systems for carbohydrate valorization. Overall, the combination of competitive productivity at short residence times in a low-hazard solvent system positions these niobium-based catalysts favorably relative to many reported heterogeneous HMF routes that rely on longer reaction times and less sustainable solvents. Moreover, the pelletized catalyst format is directly compatible with fixed-bed continuous-flow reactors, facilitating catalyst handling, separation, and regeneration.

## Graphical abstract

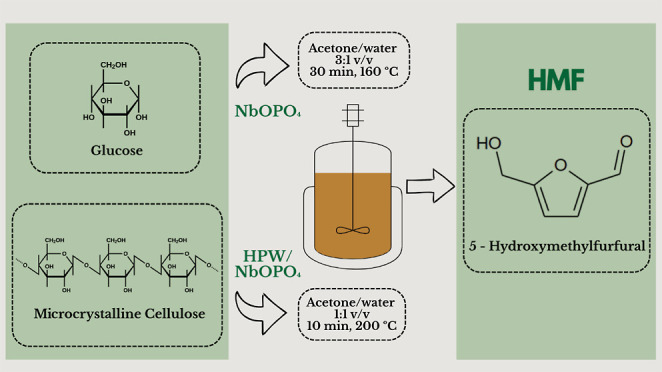

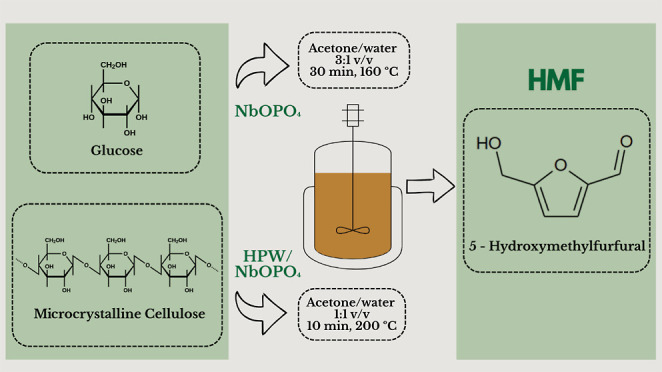

## 1 Introduction

The growing demand for renewable energy sources and sustainable materials has intensified research into biorefineries based on lignocellulosic biomass for the production of high-value chemicals.^1,2^ Among the target compounds, 5-hydroxymethylfurfural (HMF) stands out as a key platform molecule, identified by the U.S. Department of Energy as one of the top ten value-added chemicals derived from biomass.^3,4^ HMF serves as a versatile intermediate in the synthesis of fine chemicals, polymeric materials, biofuels, pharmaceuticals, and agrochemicals.^5^ Notably, its derivatives include 2,5-dimethylfuran (DMF), a promising biofuel, and 2,5-furandicarboxylic acid (FDCA), a monomer used in the production of polyethylene furanoate (PEF), a biobased alternative to polyethylene terephthalate (PET).^6–8^

Beyond its academic relevance, the HMF–FDCA–PEF value chain has recently gained clear industrial momentum. Notably, Avantium has reported the official opening and commissioning progress of its FDCA flagship plant in Delfzijl (The Netherlands), designed to produce up to 5 kt·year^–1^ of FDCA, underscoring the transition from pilot demonstrations to commercial-scale implementation.^9^ In parallel, recent reviews emphasize that the 5-HMF route has received significant attention and is expected to play a leading role in the industrial production of FDCA, reinforcing the need for robust, scalable, and greener HMF manufacturing technologies.^10^

HMF production occurs through acid-catalyzed dehydration of carbohydrate feedstocks, including monomeric hexoses (fructose and glucose), polysaccharides (notably cellulose), and lignocellulosic biomass. Although fructose generally affords higher HMF yields, glucose has attracted growing interest due to its lower cost and greater natural abundance. Cellulose, despite its structural complexity as a glucose-based polysaccharide, has gained increasing attention because it does not compete with food resources, unlike glucose and fructose, and is more abundant due to its high proportion in lignocellulosic biomass.^11^

Given the differing reactivities of these feedstocks, the selection of catalysts is a determining factor for achieving efficient HMF production. Both homogeneous and heterogeneous acid catalysts have been investigated, including inorganic acids (e.g., HCl, H_2_SO_4_), metal chlorides (e.g., FeCl_3_, RuCl_3_), metal oxides (e.g., Nb_2_O_5_, Al_2_O_3_), and zeolites.^4,7,12–15^

Homogeneous catalysts generally provide high HMF yields, with values exceeding 40% from glucose^14,16–18^ and 30% from commercial cellulose.^13,14,19^ However, industrial application of these catalysts is hindered by drawbacks such as equipment corrosion, environmental concerns related to waste disposal, challenging product separation, and high purification costs.^20,21^ To avoid these issues, heterogeneous catalysts present an attractive alternative as they generally allow for easier product separation, catalyst recovery and reuse, resulting in lower process costs.^22,23^

Heterogeneous catalysts have shown promising results in the conversion of glucose to HMF, with yields higher than 60%.^15,24–29^ This performance is often associated with the presence of both Lewis and Brønsted acid sites within the catalyst structure. Lewis acid sites catalyze the isomerization of glucose to fructose, while Brønsted acid sites catalyze the subsequent dehydration of fructose to HMF, resulting in the enhanced overall efficiency.^12,24^

In cellulose conversion, heterogeneous catalysts typically achieve HMF yields between 30% and 50%,^8,20,29–31^ which are generally lower than those obtained with metal chloride catalysts (∼50–80%).^13,19,32^ This performance gap is often attributed to mass transfer limitations resulting from the low solubility of cellulose in common solvents and consequently reduced contact with solid catalysts. Nevertheless, heterogeneous catalysts produce higher amounts of HMF than homogeneous Brønsted acid systems, which typically achieve only 10–15% yields due to increased humin formation.^30,33^

Despite the promising results reported for heterogeneous catalysts in HMF production, developing systems that remain stable and reusable under aqueous or water-containing reaction media remains challenging. Catalyst deactivation caused by humin deposition on the surface is frequently observed, often requiring calcination between cycles and limiting long-term applicability.^4,34^ In addition, maintaining structural integrity and catalytic activity in water-containing systems is particularly important as many HMF production processes are conducted in aqueous, biphasic, or mixed solvent environments.^15,27,30,31,34^

In this context, niobium-based catalysts such as Nb_2_O_5_ and NbOPO_4_ have attracted attention due to their desirable acidic strength, tunable Brønsted/Lewis acidity, and good stability under hydrothermal conditions. Notably, their Lewis acid sites can remain active even in the presence of water coordination, making them suitable for reactions conducted in water-containing media.^35,36^ Furthermore, the incorporation of strong Brønsted acids such as phosphotungstic acid (HPW) can enhance dehydration efficiency, particularly in the conversion of microcrystalline cellulose, where stronger Brønsted acidity is required to promote hydrolysis and subsequent dehydration steps.^37^

Given the relevance of solvent selection for ensuring safety, health, and environmental compliance, the choice of reaction medium is a decisive factor in advancing sustainable catalytic processes. Although polar aprotic solvents such as dimethyl sulfoxide (DMSO) and tetrahydrofuran (THF) are commonly reported in the literature for HMF production reactions,^8,15,29,30,38,39^ both are classified as ‘problematic’ according to the CHEM21 solvent selection guide,^40^ mainly due to concerns related to toxicity, environmental persistence, and occupational hazards. In this context, acetone emerges as a favorable alternative, being a nontoxic, readily biodegradable solvent, and is classified as “recommended” by the same guide. Furthermore, its potential for recovery and reuse aligns with circular economy principles, contributing to the development of greener biomass conversion processes.

Although the yield is widely used in the literature as the main parameter for evaluating catalytic performance, it does not fully represent catalyst efficiency, particularly in the context of industrial-scale applications. In this regard, two complementary parameters are especially valuable: the volumetric rate, which reflects the amount of product formed over time within a given reaction volume, and the specific rate, which relates the amount of HMF produced to the mass of catalyst used per unit time. Together, these metrics provide a more realistic estimate of the process scalability. In this context, the present study investigated HMF production from glucose and microcrystalline cellulose using different niobium-based catalytic systems (NbOPO_4_ and Nb_2_O_5_), in powder and pellet forms, with and without phosphotungstic acid (HPW) impregnation. The kinetic profiles and stability of the selected catalyst for glucose conversion to HMF were also evaluated. The findings provide insights into the catalytic behavior of niobium-based systems and their potential for scalable HMF production.

## 2 Experimental Section

### 2.1 Catalysts Preparation

The catalysts niobium pentoxide (Nb_2_O_5_, HY-340) and niobium phosphate (NbOPO_4_) were provided by the Companhia Brasileira de Metallurgia e Mineração (CBMM). Nb_2_O_5_ and NbOPO_4_ powders were calcined at 250 °C for 3 h (Figure 1a). The pellet catalysts were prepared by adding 25% (w/v) powder catalyst (Nb_2_O_5_ or NbOPO_4_) and 140 mL of oxalic acid solution (10% w/v) to a 200 mL stainless-steel autoclave reactor. The sealed reactor was heated in an oven at 175 °C for 8 h. The material was then vacuum-filtered, and the resulting cake was dried in an oven at 50 °C for 48 h. To obtain the pellets, the material was mixed with a 5% (w/v) oxalic acid solution until a moldable mass was formed. This mass was extruded to produce cylindrical pellets with a diameter of 2 mm and a length of 5 mm, as shown in Figure 1b. Finally, the pellets were calcined at 250 °C for 3 h. The pelletized morphology was selected to provide a structured and recoverable catalyst form, particularly relevant for reaction systems involving solid feedstocks while maintaining a simpler shaping approach than more engineered configurations such as monoliths or beads.

**Figure 1:**
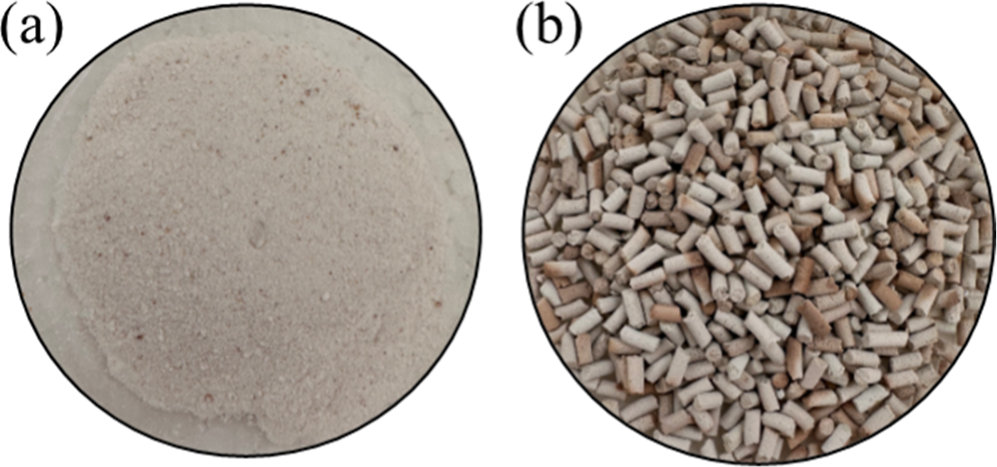
NbOPO_4_ catalyst in the powder form (a) and pellet form (b).

For the individual catalysts, heterogeneous catalysts were prepared by combining phosphotungstic acid (HPW) with Nb_2_O_5_ or NbOPO_4_, either in powder or pellet form, through an impregnation method. The HPW/Nb_2_O_5_ and HPW/NbOPO_4_ powder catalysts were prepared using the incipient wetness impregnation method, as described in a previous work.^41^ In this procedure, the supports (Nb_2_O_5_ or NbOPO_4_), previously calcined at 250 °C for 3 h, were impregnated with a 70% ethanol solution containing the active phase (HPW). This impregnation step was repeated three times until the desired metal loading of 30% (w/w) was achieved. The selection of this loading was based on the need to ensure sufficient Brønsted acidity while preserving the structural integrity and textural properties of the support since higher HPW loadings have been reported to promote agglomeration of Keggin units and partial pore blockage, resulting in decreased surface area and pore volume and ultimately limiting the effective utilization of HPW.^42^ After impregnation, the catalysts were dried at 100 °C for 2 h and then calcined at 250 °C for 3 h.

For the HPW/Nb_2_O_5_ and HPW/NbOPO_4_ pellet catalysts, a wet impregnation method was employed as the stirring required for the incipient wetness impregnation could potentially damage the pellets. In the wet impregnation process, the supports (Nb_2_O_5_ or NbOPO_4_), also calcined at 250 °C for 3 h, were impregnated with 100 mL of a 10% (w/v) HPW solution, with an ethanol-to-water ratio of 7:3 (v/v), to achieve a 30% (w/w) metal loading. The mixture was agitated in a rotary evaporator at room temperature with a rotation of 20 rpm for 5 h. The solvent was removed from the material by vacuum evaporation in a rotary evaporator at 60 °C. Finally, the catalysts were dried in an oven at 100 °C for 6 h and calcined at 250 °C for 3 h.

### 2.2 HMF Catalytic Synthesis

The production of HMF from glucose and microcrystalline cellulose was investigated using different catalytic systems: hybrid, impregnated, and individual. In these experiments, Nb_2_O_5_ and NbOPO_4_, in both powder and pellet forms, were combined with HPW in a hybrid system, where HPW was dissolved in the reaction medium; or in an impregnated system, where HPW was impregnated on the surface of Nb_2_O_5_ or NbOPO_4_. The catalysts were also employed individually, representing each catalytic system. Table 1 presents the catalytic systems, strategies, and loadings of the different catalysts employed in the experiments.

**Table 1:** Catalytic Systems, Strategies, and Loadings of the Different Catalysts Used in the HMF Production from Glucose and Microcrystalline Cellulose

Exp	Catalyst	Catalytic system	Loading (g)	Catalytic strategy
1	No catalyst	-	-	-
2	HPW	Individual	1.5	Homogeneous
3	NbOPO_4_ powder	Individual	3.5	Heterogeneous
4	Nb_2_O_5_ powder	Individual	3.5	Heterogeneous
5	NbOPO_4_ pellet	Individual	3.5	Heterogeneous
6	Nb_2_O_5_ pellet	Individual	3.5	Heterogeneous
7	HPW + NbOPO_4_ powder	Hybrid	5.0 (1.5 HPW +3.5 NbOPO_4_)	Mixed
8	HPW + Nb_2_O_5_ powder	Hybrid	5.0 (1.5 HPW +3.5 Nb_2_O_5_)	Mixed
9	HPW + NbOPO_4_ pellet	Hybrid	5.0 (1.5 HPW +3.5 NbOPO_4_)	Mixed
10	HPW + Nb_2_O_5_ pellet	Hybrid	5.0 (1.5 HPW +3.5 Nb_2_O_5_)	Mixed
11	HPW/NbOPO_4_ powder	Impregnated	5.0 (30 wt % HPW/NbOPO_4_)	Heterogeneous
12	HPW/Nb_2_O_5_ powder	Impregnated	5.0 (30 wt % HPW/Nb_2_O_5_)	Heterogeneous
13	HPW/NbOPO_4_ pellet	Impregnated	5.0 (30 wt % HPW/NbOPO_4_)	Heterogeneous
14	HPW/Nb_2_O_5_ pellet	Impregnated	5.0 (30 wt % HPW/Nb_2_O_5_)	Heterogeneous

The reactions were conducted in duplicate using pressurized stainless-steel reactors (Parr series 4566) containing 100 mL of reaction medium under an agitation of 500 rpm. The reaction conditions for glucose consisted of a 50 g/L substrate in an acetone-to-water mixture (1:1 v/v) at 160 °C for 30 min. For microcrystalline cellulose, the conditions consisted of a 10% (w/v) substrate in an acetone-to-water mixture (3:1 v/v) at 200 °C for 10 min. These conditions were established based on prior optimization studies carried out in our research group employing other niobium-based catalytic systems for glucose^41^ and microcrystalline cellulose.^43^ Based on these experiments, the most effective catalytic systems were identified for each feedstock, enabling a comparative analysis of catalyst performance in the conversion of monomeric (glucose) and polymeric (microcrystalline cellulose) substrates. The reported catalytic performance values correspond to the mean of duplicate experiments, and the error bars represent standard deviations.

In the subsequent stage, the kinetic profile of the selected catalyst for the conversion of glucose to HMF was evaluated. At this stage, a new batch of niobium phosphate was employed. Reactions were carried out under the same conditions used for HMF production from glucose, employing 3.5% (w/v) of the catalyst in either powder or pellet form. The kinetic study was performed by using reaction times ranging from 0 to 120 min, and the optimal reaction time for each catalyst form was defined as the time at which approximately 80% glucose conversion was achieved.

The concentration profiles of glucose (G), 5-hydroxymethylfurfural (HMF, H), furfural (F), and byproducts (Bp) from the kinetic experiments were described by using a lumped reaction network with catalyst deactivation. The term Bp represents byproducts formed during the reaction, such as compounds derived from HMF rehydration, including levulinic acid and formic acid as well as humins. The following pathways were considered: *G* → *H*, *H* → *F*, *G* → Bp, and *H* → Bp. All reaction steps were assumed to follow apparent first-order kinetics, with respect to the reacting species concentration.

To account for progressive catalyst activity loss during reaction, a time-dependent activity function *a*(*t*) was included as a multiplicative term in all rate expressions. Catalyst deactivation was modeled as first-order, as described in eq 1.

1 $$\frac{da}{dt}=-k_{d}a,a\left(0\right)=1$$

leading to *a*(*t*) = exp­(−*k*_*d*_ *t*), where *k*_*d*_ is the deactivation constant.

The model equations are given by eqs 2–5

2 $$\frac{dG}{dt}=-\left(k_{1}+k_{3}\right)a\left(t\right)G$$

3 $$\frac{dH}{dt}=k_{1}a\left(t\right)G-\left(k_{2}+k_{4}\right)a\left(t\right)H$$

4 $$\frac{dF}{dt}=k_{2}a\left(t\right)H$$

5 $$\frac{dBp}{dt}=k_{3}a\left(t\right)G+k_{4}a\left(t\right)H$$

Initial conditions were taken from experimental measurements at *t* = 0: *G*(0) = *G*_0_, *H*(0) = *H*_0_, *F*(0) = *F*_0_, and Bp(0) = Bp_0_. The unknown parameters (*k*_1_,*k*_2_,*k*_3_,*k*_4_,*k*_*d*_) were estimated by nonlinear least-squares regression, as defined in eq 6, minimizing the weighted sum of squared residuals between experimental and model-predicted concentrations for all species and sampling times.

6 $$\min\sum_{i}\left[w_{G}{\left(G_{i}^{\exp}-G_{i}^{\mathrm{mod}}\right)}^{2}+w_{H}{\left(H_{i}^{\exp}-H_{i}^{\mathrm{mod}}\right)}^{2}+w_{F}{\left(F_{i}^{\exp}-F_{i}^{\mathrm{mod}}\right)}^{2}+{,w}_{Bp}{\left(Bp_{i}^{\exp}-Bp_{i}^{\mathrm{mod}}\right)}^{2}\right]$$

Parameter estimation was performed in Microsoft Excel using the Solver add-in (GRG Nonlinear algorithm), with non-negativity constraints imposed on all kinetic and deactivation constants. Model adequacy was assessed by residual analysis and goodness-of-fit metrics (e.g., *R* ^2^ and RMSE) for each species.

Following the kinetic study, the catalyst performance in consecutive HMF production cycles was then investigated through a recycling study comprising 11 successive reaction runs. The reaction time was set at 30 min for the powder catalyst and 90 min for the pellet according to the kinetic data. After each cycle, the catalyst was recovered and reused without any intermediate treatment—no washing or thermal treatment was applied between reactions.

### 2.3 Analytical Methods

The crystalline structure of the heterogeneous catalysts previously selected in the HMF production stage from glucose and microcrystalline cellulose was analyzed by using X-ray powder diffraction (XRD). The measurements were performed on a PANalytical Empyrean diffractometer with Cu Kα radiation (λ = 1.5418 Å) operating at 40 kV and 30 mA. The scanning range was set from 10° to 90°, with a step size of 0.02° and a counting time of 50 s per step.

The textural properties of the catalysts were analyzed via N_2_ adsorption by using a Quantachrome NOVA 2200e instrument. Prior to the analysis, 0.2 g of the sample was placed in a glass cell and heated at 200 °C for 2 h under vacuum to eliminate any surface-adsorbed impurities. The specific surface area was determined using the BET method (Brunauer–Emmett–Teller), while the pore volume and pore diameter were calculated using the BJH method (Barrett–Joyner–Halenda).

The different types of acid sites (Lewis and Brønsted) in the catalysts were qualitatively analyzed by using attenuated total reflection Fourier-transform infrared spectroscopy (ATR-FTIR) with pyridine adsorption. Approximately 30 mg of each sample were weighed and pretreated under a nitrogen flow of 100 mL/min at 200 °C for 2 h to remove water and adsorbed gases from the surface and pores of the catalysts. The samples were then exposed to pyridine vapor carried by nitrogen at 150 °C for 1 h and 30 min. Afterward, the catalysts were purged with nitrogen at 150 °C for 1 h to eliminate the physisorbed pyridine. The spectra were acquired with a resolution of 4 cm^–1^ and 64 scans per sample, covering the spectral range of 4000 to 600 cm^–1^. The spectra of pyridine-adsorbed catalysts were obtained by using the untreated catalysts as the background.

The thermal stability of the catalysts, both before and after the reaction, was assessed through thermogravimetric analysis (TGA), which was conducted by using a Shimadzu TGA 50 instrument. The analysis was performed under the following conditions: a nitrogen flow rate of 50 mL/min, a heating rate of 10 °C/min, and a temperature range from 30 to 1000 °C.

The glucose concentration was determined using high-performance liquid chromatography (HPLC) on a Shimadzu system equipped with an isocratic pump, a refractive index detector, and a Bio-Rad Aminex HPX-87H column (300 × 7.8 mm). The analysis was performed under the following conditions: column temperature of 45 °C, 0.005 mol/L sulfuric acid solution as the mobile phase, flow rate of 0.6 mL/min, and sample injection volume of 0.02 mL. Similarly, the HMF concentration was determined by HPLC but using an UV detector set to 276 nm and a Waters Spherisorb C18 column (100 × 4.6 mm, 5 μm particle size). The analysis was conducted at room temperature with a mobile phase consisting of a 1:8 (v/v) acetonitrile-to-water ratio containing 1% acetic acid, a flow rate of 0.8 mL/min, and a sample injection volume of 0.02 mL.

The cellulose concentration was determined by converting cellulose into glucose, which was then quantified by high-performance liquid chromatography (HPLC). The first step was carried out following a procedure adapted from the National Renewable Energy Laboratory.^44^ After the HMF production reaction, the remaining cellulose was separated from the reaction medium by centrifugation and dried at 105 °C until a constant mass was reached. Subsequently, 10 mL of 72% (w/w) H_2_SO_4_, preheated to 45 °C, was added to a beaker containing 1.5 g of the dry cellulose, and the mixture was maintained in a thermostatic bath at 45 °C for 7 min with constant agitation. The mixture was then diluted in 275 mL of distilled water in a 500 mL Erlenmeyer flask, which was capped with aluminum foil and autoclaved at 121 °C for 45 min. After being autoclaved, the material was filtered and diluted in a 500 mL volumetric flask. The glucose concentration was then determined by HPLC, as previously described.

Glucose conversion (*X*_Glu_), cellulose conversion (*X*_Cel_), HMF yield (*Y*_HMF_), and HMF selectivity (*S*_HMF_) were calculated according to eqs 7–10, with all concentrations expressed in mol/L.

7 $$X_{Glu}\left(\%\right)=\frac{{\left[Glucose\right]}_{initial}-{\left[Glucose\right]}_{final}}{{\left[Glucose\right]}_{initial}}\cdot 100$$

8 $$X_{Cel}\left(\%\right)=\frac{{\left[Cellulose\right]}_{initial\left(based\;on\;glucose\;unit\right)}-{\left[Glucose\right]}_{after\;conversion}}{{\left[Cellulose\right]}_{initial\left(based\;on\;glucose\;unit\right)}}\cdot 100$$

$$Y_{HMF}\left(\%\right)=\frac{{\left[HMF\right]}_{produced}}{{\left[Glucose\,\;\;or\;\;cellulose\right]}_{initial}}\cdot 100$$ 9

10 $$S_{HMF}\left(\%\right)=\frac{Y_{HMF}}{X_{Glu}\;\mathrm{or}\;X_{Cel}}\cdot 100$$

## 3 Results and Discussion

### 3.1 HMF Production from Glucose

The results of HMF production from glucose using different catalytic systems (hybrid, impregnated, and individual) are shown in Figure 2. HMF concentrations ranged from 0.18 to 10.74 g/L, corresponding to yields between 0.53% and 30.70%. The highest HMF concentrations and yields, above 9 g/L and 26%, respectively, were obtained with NbOPO_4_ and Nb_2_O_5_ in the powder form, irrespective of their configuration as individual, hybrid, or impregnated catalysts. This superior performance is likely due to the greater accessibility and improved homogenization of the catalytic system provided by the powdered form, in contrast to pellet catalysts, which tend to hinder the diffusion of reactant and product molecules.

**Figure 2:**
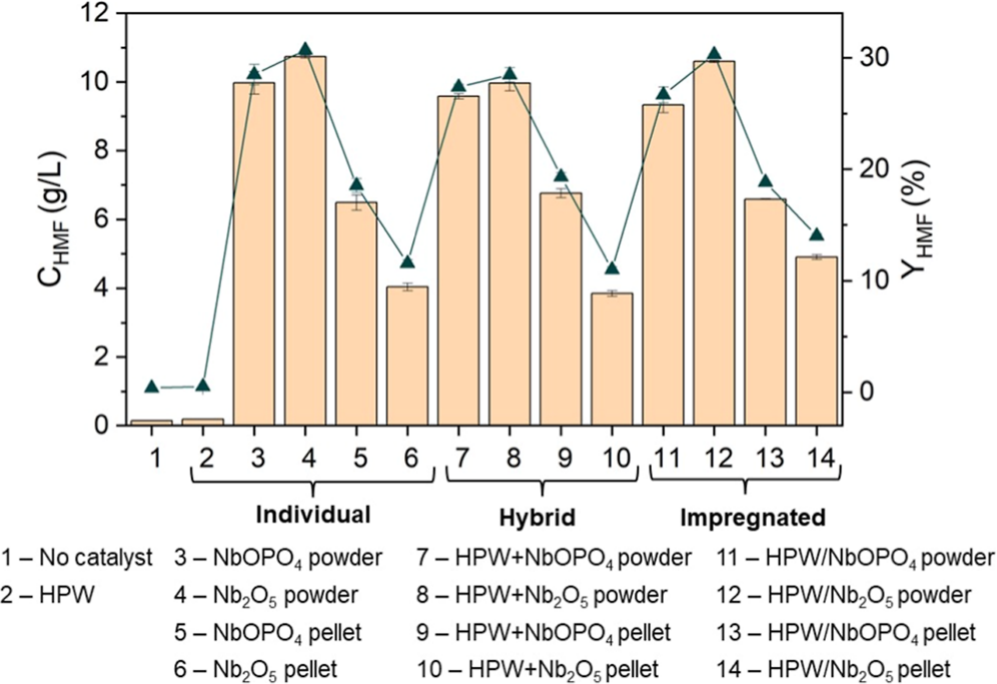
HMF concentration (*C*_HMF_, bars) and yield (*Y*_HMF_, line) from glucose using different catalytic systems. All reactions were conducted using 50 g/L glucose, an acetone-to-water ratio of 1:1 (v/v), a reaction medium of 100 mL, stirring at 500 rpm, at 160 °C for 30 min. Data represent mean values of duplicate experiments; error bars indicate standard deviations.

It is worth highlighting that HPW exhibited no catalytic activity, when used either individually or in combination with other catalysts. Although HPW is commonly employed to enhance Brønsted acidity—an essential requirement for the dehydration of fructose into HMF following the glucose-to-fructose isomerization step—its contribution was negligible under the investigated conditions. This finding indicates that NbOPO_4_ and Nb_2_O_5_ alone possess sufficient acidity to promote the glucose-to-HMF transformation.

Selectivity was also evaluated for HMF production from glucose (Figure 3), with results ranging from 5.57 to 39.55%. The highest selectivity values (37.29–39.66%) were achieved by the niobium phosphate-based catalysts, regardless of their catalytic system or physical form. Niobium oxide-based catalysts, on the other hand, exhibited selectivity values between 18.44 and 32.04%. For these catalysts, the powder form displayed approximately 73% higher selectivity than the pellet form, indicating that for Nb_2_O_5_, the powder is more effective than the pellet.

**Figure 3:**
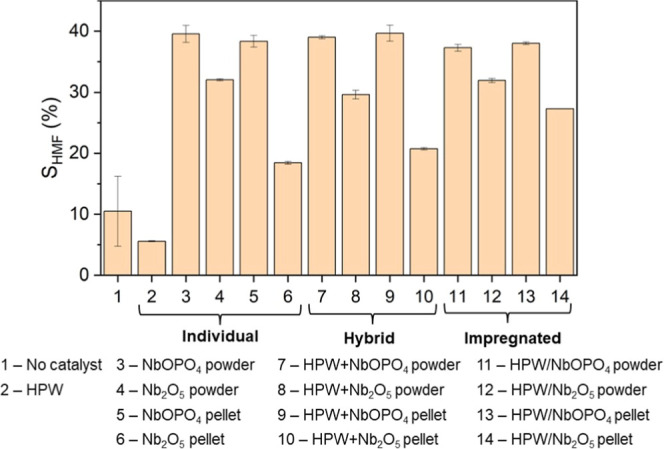
HMF selectivity (*S*_HMF_) from glucose using different catalytic systems. All reactions were conducted using 50 g/L glucose, an acetone-to-water ratio of 1:1 (v/v), a reaction medium of 100 mL, stirring at 500 rpm, at 160 °C for 30 min. Data represent mean values of duplicate experiments; error bars indicate standard deviations.

By combination of the results shown in Figures 2 and 3, NbOPO_4_ powder was selected as the most effective catalyst for glucose conversion into HMF as it provided the best combination of concentration (9.98 g/L), yield (28.51%), and selectivity (39.55%). It is worth noting, however, that the NbOPO_4_ pellet exhibited selectivity values similar to the powdered catalyst but with lower HMF concentration and yield. This suggests that extending the reaction time could allow the pelletized form to achieve performance comparable to the powdered catalyst. Considering its practical advantage of easier recovery and reuse, the pellet was also selected for further investigation. Therefore, in the next stage, the kinetic profiles of both powder and pellet forms of NbOPO_4_ were examined together with their reusability in consecutive cycles of glucose conversion into HMF.

#### 3.1.1 Kinetic Profile Evaluation of the HMF Production from Glucose Using NbOPO_4_

The kinetic profile of glucose conversion to HMF was investigated by using NbOPO_4_ catalysts in both powder and pellet forms (Figure 4), which had been previously selected. Within the studied interval of 120 min, the maximum HMF concentration and yield obtained with NbOPO_4_ powder were 14.67 g/L and 41.9% at 45 min, while for the pellet, they reached 12.48 g/L and 35.7% at 120 min. These results confirmed the hypothesis that longer reaction times allow the pellet to achieve performance comparable to the powdered catalyst. The difference in reaction time required is most probably related to the larger surface area of the powder catalyst. This limitation could be mitigated by increasing the mass of the pellet catalyst, a strategy justified by its easier recovery and reuse.

**Figure 4:**
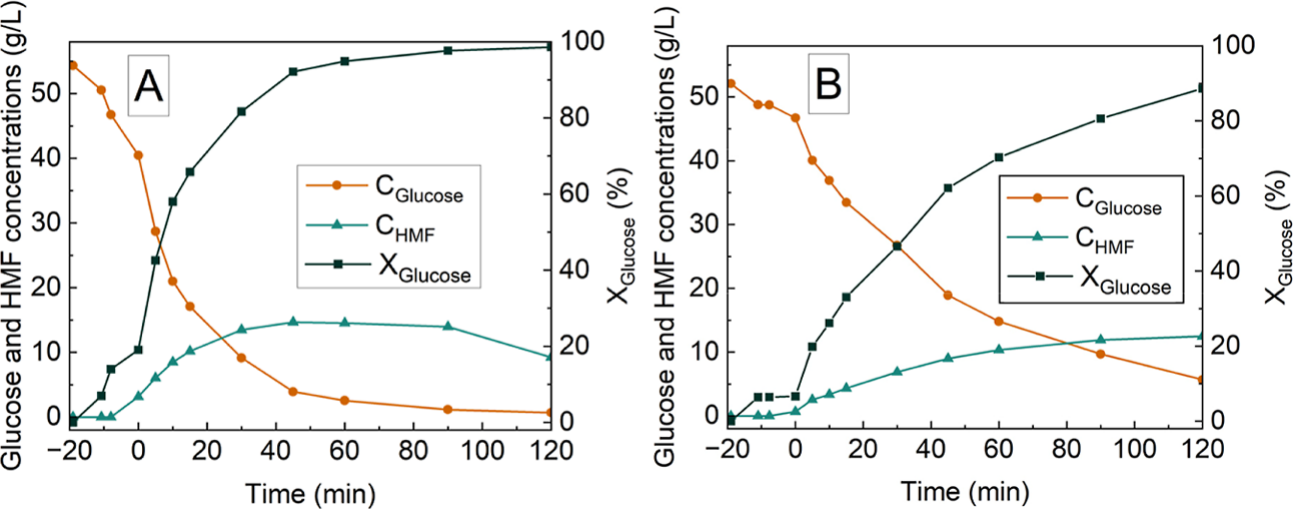
Kinetic profiles of HMF production from glucose using NbOPO_4_ in the powder form (A) and pellet form (B) as catalysts, showing HMF concentration (*C*_HMF_), glucose concentration (*C*_Glucose_), and glucose conversion (*X*_Glucose_) as a function of reaction time. All reactions were conducted with 50 g/L glucose, 3.5% (w/v) catalyst loading, an acetone-to-water ratio of 1:1 (v/v), a total reaction volume of 100 mL, stirring at 500 rpm, at 160 °C for up to 120 min. *Negative time values represent the reactor heating period.

In addition, the kinetic study was used to define the reaction times applied in the subsequent evaluation of catalyst reusability in consecutive cycles of glucose conversion into HMF. The reaction time was set at the point corresponding to approximately 80% glucose conversion for each catalyst form: 30 min for the powder and 90 min for the pellet (Table 2). This criterion was chosen to ensure a balance between conversion and volumetric reaction rates while maintaining high HMF selectivity and minimizing the formation of degradation products. Table 2 summarizes the kinetic parameters that support this analysis. It also provided a standardized basis for the subsequent reusability evaluation of both catalyst forms.

**Table 2:** Summary of Process Parameters for Glucose Conversion into HMF using NbOPO_4_ Catalysts in Powder and Pellet Forms^a^

Parameters	NbOPO_4_ powder (30 min)	NbOPO_4_ powder (45 min)	NbOPO_4_ pellet (90 min)	NbOPO_4_ pellet (120 min)
HMF concentration (g/L)	13.46	14.67	11.92	12.48
HMF yield (%)	38.46	41.91	34.06	35.66
Glucose conversion (%)	81.68	92.16	80.60	88.62
HMF selectivity (%)	47.08	45.48	42.25	40.24
Volumetric rate, Q (g/L·h)	26.92	19.56	7.95	6.24

a All reactions were conducted using 50 g/L glucose, 3.5% (w/v) catalyst loading, an acetone-to-water ratio of 1:1 (v/v), a reaction medium of 100 mL, stirring at 500 rpm, at 160 °C for 30 min.

To complement the descriptive kinetic analysis, a global kinetic model was developed for both NbOPO_4_ catalysts (powder and pellet), as shown in Figure 5, to estimate the apparent kinetic parameters associated with the global reaction network. In the powder-catalyst system (Figure 5A), the kinetic profiles show the expected sequential behavior: fast glucose depletion, transient HMF accumulation followed by partial decline, and progressive byproduct formation, while furfural remains at low concentrations throughout the experiment. As summarized in Table 3, the powder data set presented strong agreement between model and experiments, particularly for glucose and byproducts (*R*_1‑SSE/SST_^2^ = 0.9976 and 0.9755, respectively), with moderate performance for HMF (0.8948) and comparatively lower accuracy for furfural (0.5095), consistent with the low absolute concentration range of this species. The same trend is visually confirmed by the close overlap between fitted curves and experimental points in Figure 5A, especially in the early time glucose decay and late-time byproduct growth regions.

**Figure 5:**
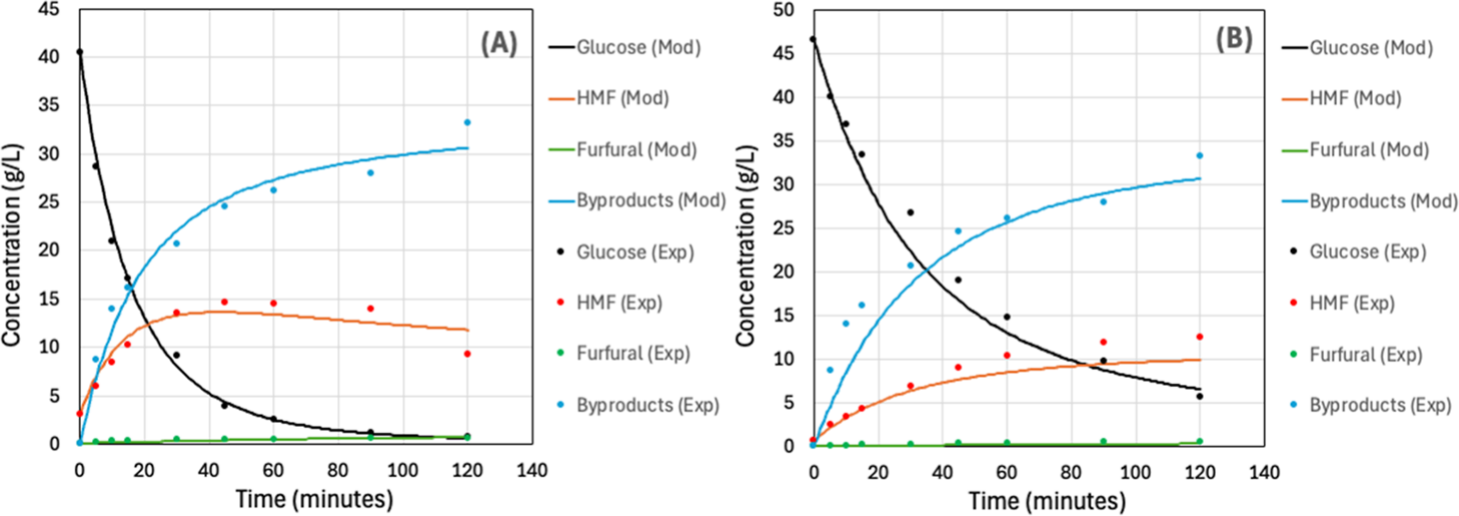
Kinetic modeling of HMF production from glucose using NbOPO_4_ in the powder form (A) and pellet form (B) as catalysts. All reactions were conducted with 50 g/L glucose, 3.5% (w/v) catalyst loading, an acetone-to-water ratio of 1:1 (v/v), a total reaction volume of 100 mL, stirring at 500 rpm, at 160 °C for up to 120 min.

**Table 3:** Model Fitting Quality (Powder vs Pellets)

Metric	Powder	Pellets
*R* ^2^—Glucose (G)	0.9976	0.9766
*R* ^2^—HMF (H)	0.8948	0.8782
*R* ^2^—Furfural (F)	0.5095	0.0753
*R* ^2^—Byproducts (Bp)	0.9755	0.9104
Total SSE (objective function)	40.339	136.766

When both catalyst morphologies are compared (Figure 5A, B; Tables 3 and 4), powder provides a substantially lower global residual error (total SSE = 40.339) than pellets (136.766), indicating a better overall fitting performance under identical operating conditions. This difference is also reflected in the estimated rate constants (Table 4), with higher apparent values for powder in the main pathways (*k*_1_, *k*_2_, and *k*_3_) and similar deactivation constants (*k*_*d*_). In contrast, the pellet case (Figure 5B) shows slower apparent conversion dynamics and *k*_4_ → 0, suggesting reduced identifiability of secondary routes, likely due to stronger mass-transfer limitations (external film and intraparticle diffusion). Overall, these results indicate that powder behaves closer to reaction-controlled kinetics, whereas pellets operate under a more pronounced mixed kinetic–diffusional regime.

**Table 4:** Estimated Kinetic Constants and Powder-to-Pellet Ratios

Parameter	Powder	Pellets	Ratio (powder/pellets)
k1	0.02370	0.006731	3.52
k2	0.000760	0.000514	1.48
k3	0.03931	0.02185	1.80
k4	0.007249	0.000000	—^a^
kd	0.01099	0.01034	1.06

a Not computed because *k*_4_ = 0 for pellets.

#### 3.1.2 Catalyst Performance in Consecutive HMF Production Cycles

The catalytic performance of NbOPO_4_ (powder and pellet forms) for HMF production from glucose was evaluated over 11 consecutive reaction cycles conducted without any thermal treatment or washing of the catalyst between runs. The reactions were carried out using the previously defined times of 30 min for the powder and 90 min for the pellet. Figure 6 presents the results of catalyst recycling for NbOPO_4_ in powder and pellet forms. In general, HMF production using the powder catalyst exhibited greater stability over consecutive cycles compared with the pellet form. For the powder, HMF concentration, yield, and selectivity remained high and relatively constant up to the seventh cycle, with variations of approximately 14% for concentration and yield and 7% for selectivity. In contrast, for the pellet catalyst, these parameters remained elevated and stable only up to the fourth cycle, with variations of approximately 14% for concentration and yield and 11% for selectivity.

**Figure 6:**
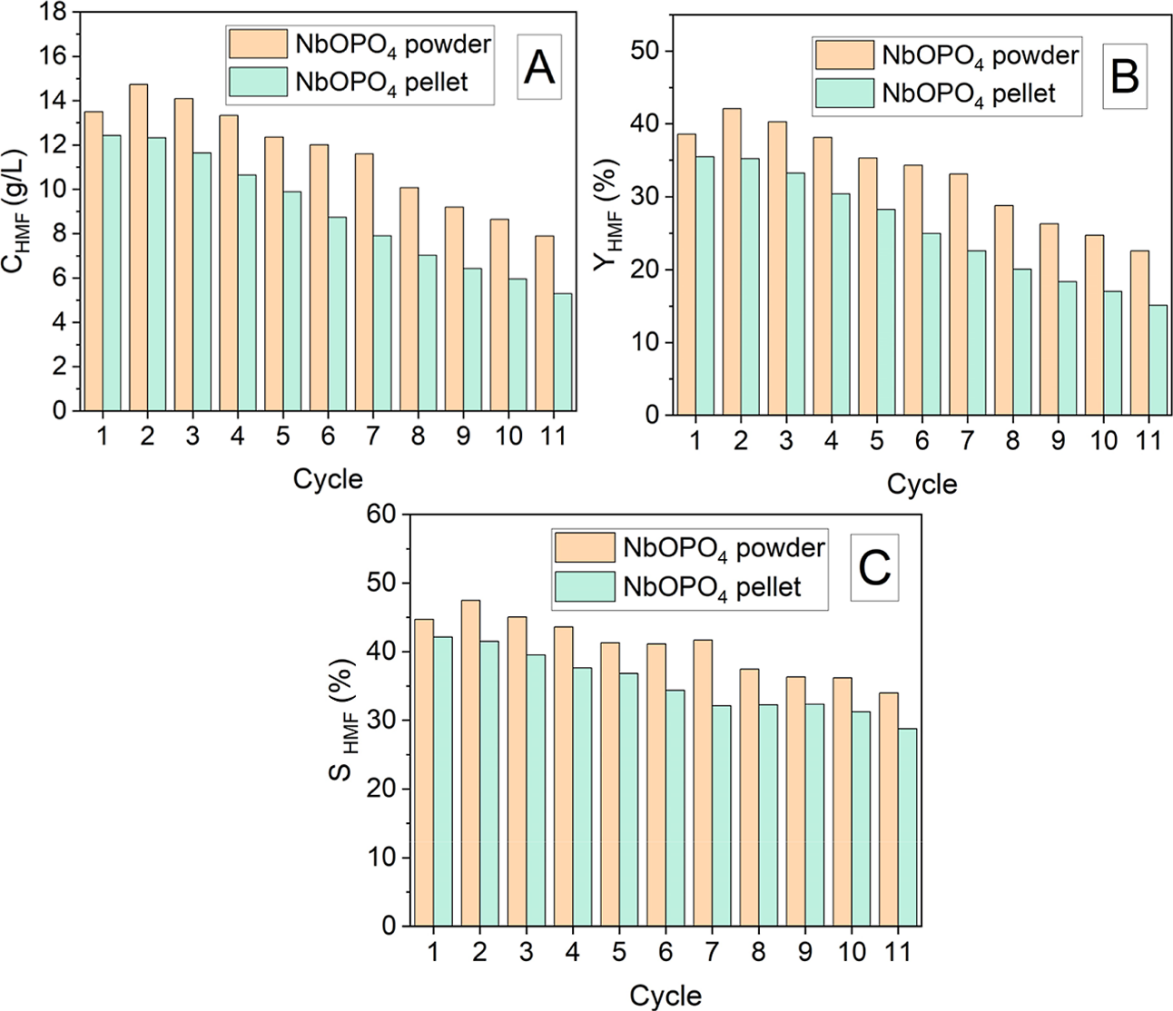
Catalytic performance of NbOPO_4_ in powder and pellet forms for HMF production from glucose over 11 consecutive reaction cycles, showing HMF concentration (*C*_HMF_), (A), yield (*Y*_HMF_), (B) and selectivity (*S*_HMF_), (C). Reactions were conducted with 50 g/L glucose, 3.5% (w/v) catalyst, acetone-to-water 1:1 (v/v), 100 mL, 500 rpm, at 160 °C for 30 min (powder) and 90 min (pellet).

These results confirm the superior performance of the NbOPO_4_ powder catalyst in consecutive HMF production cycles compared with the pellet form, demonstrating greater stability. Nevertheless, the performance of the pellet catalyst after four cycles remains relevant as this form can be more easily separated from the reaction medium and retained its macroscopic shape throughout the recycling experiments, with only minor fragmentation observed. These observations indicate preserved physical integrity under the applied reaction conditions and suggest that the pelletized form may be suitable for practical catalyst handling and further process development.

The reduction in the evaluated parameters after consecutive reaction cycles may be attributed to pore blockage caused by humin formation, which was visually evidenced by a color change in the catalyst (from white to dark brown) after the reactions (Figure 7). Another possible deactivation pathway involves the structural transformation of NbOPO_4_. However, significant structural changes under the investigated reaction conditions are unlikely. According to Nowak and Ziolek,^36^ niobium phosphate remains amorphous up to approximately 800 °C, with phase transition to crystalline NbOPO_4_ occurring at temperatures above this limit. Since the reaction and recycling experiments were conducted at much lower temperatures (160 °C), major structural modifications of NbOPO_4_ are not expected. Therefore, pore blockage caused by humin deposition remains the most plausible explanation for the observed decrease in the catalytic performance. The higher stability of the NbOPO_4_ powder catalyst may be associated with its larger surface area (162.92 m^2^/g) compared to the pellet form (97.14 m^2^/g), which is more likely to become deactivated by smaller amounts of humin.

**Figure 7:**
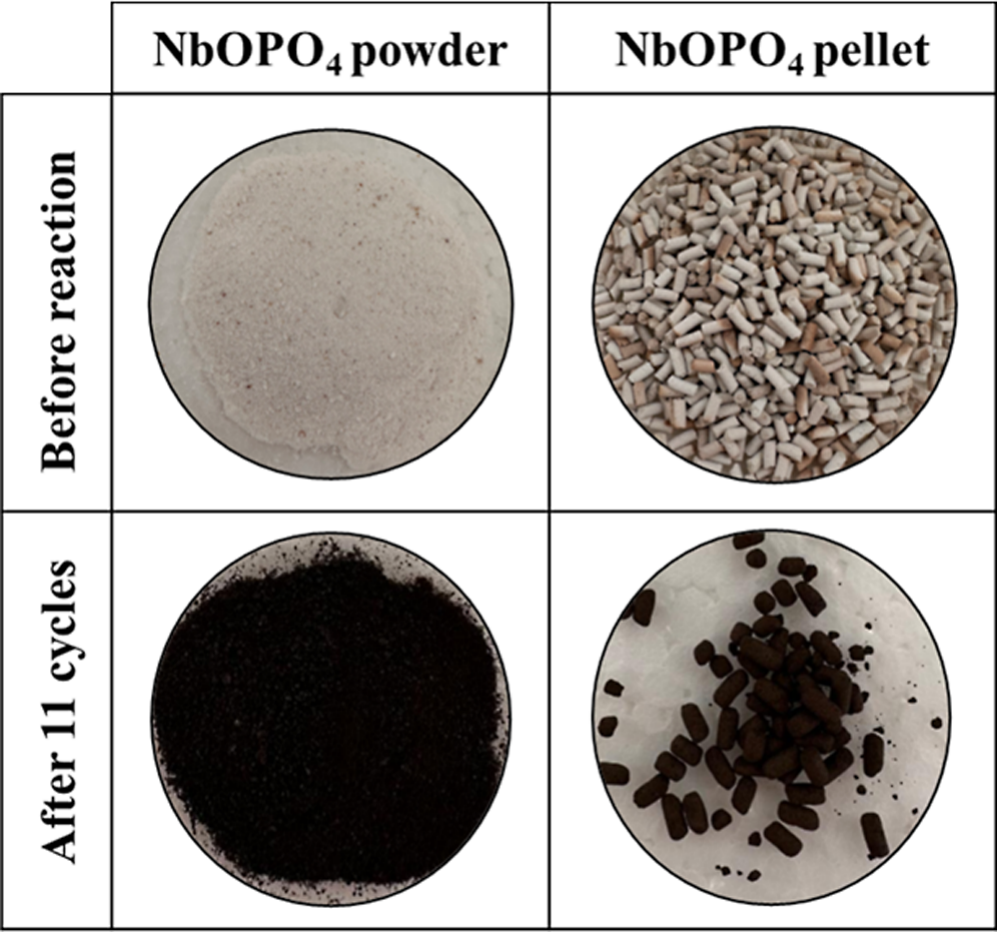
NbOPO_4_ catalysts in the powder or pellet form, before the reaction and after 11 consecutive reaction cycles.

In order to confirm the hypothesis of humin accumulation within the catalyst pores, thermogravimetric analyses were performed on NbOPO_4_ catalysts in powder and pellet forms before and after 11 reaction cycles. The resulting curves are shown in Figure 8.

**Figure 8:**
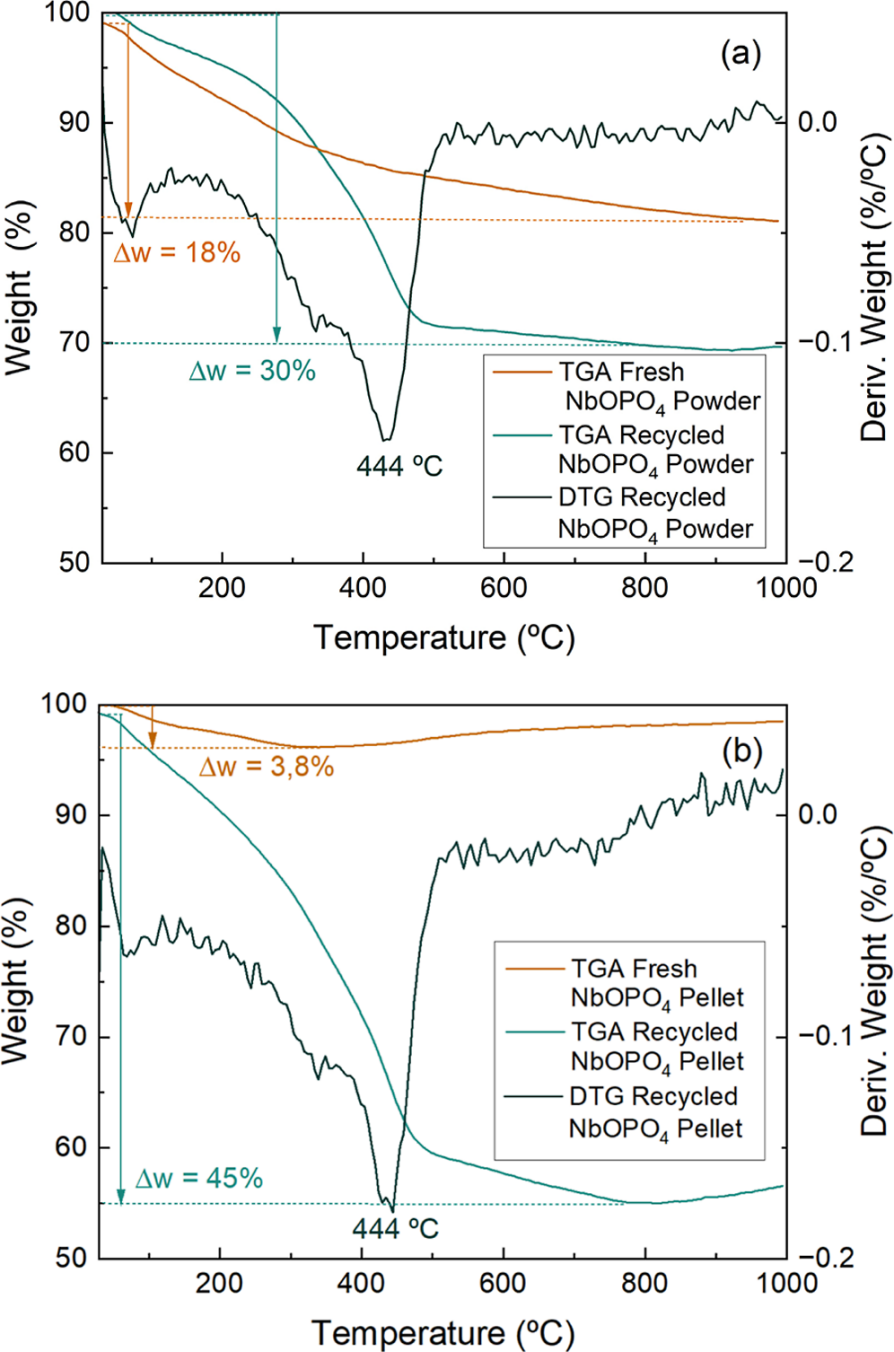
Thermogravimetric (TGA) curves of fresh and recycled NbOPO_4_ in powder (a) and pellet (b) forms, along with derivative thermogravimetric (DTG) curves of the recycled catalysts.

Thermogravimetric analysis (TGA) revealed that the maximum mass loss for the fresh catalysts was 18% for the NbOPO_4_ powder and 3.8% for the pellet. After the recycling tests, these values increased to 30% and 45%, respectively. The observed increase in mass loss, combined with the visible color change of the samples after analysis (from dark brown to white), suggests the deposition of organic matter (humin) within the catalyst pores.

The derivative thermogravimetry (DTG) curves of the catalysts after consecutive reactions exhibited a peak at 444 °C, corresponding to the stage of maximum mass loss for both materials. This peak indicates the temperature at which the removal of humin occurred.

Similarly, Candu et al.^4^ reported humin accumulation within the pores of the Nb(0.05)-Beta 18 catalyst after HMF production from glucose. Based on thermogravimetric analysis, the authors found that humin accounted for 56.4% of the catalyst mass after reaction. Furthermore, they observed that this organic matter could be removed by calcination at 450 °C, which is consistent with the findings of the present study.

These results indicate that the catalytic activity of NbOPO_4_ in powder and pellet forms could be restored through calcination at 450 °C, by removing deposited humins that may have deactivated the catalytic sites. Performing calcination after seven consecutive reactions for the powder and four reactions for the pellet could enhance the catalyst durability and reduce replacement costs.

### 3.2 HMF Production from Microcrystalline Cellulose

The results of HMF production from microcrystalline cellulose using different catalytic systems (hybrid, impregnated, and individual) are shown in Figure 9. HMF concentrations ranged from 0.08 to 14.68 g/L, corresponding to yields between 0.10% and 18.88%. The lowest values for HMF concentration and yield were observed with the individual catalysts, whether in homogeneous catalysis with HPW or in heterogeneous catalysis with NbOPO_4_ and Nb_2_O_5_ in the powder or pellet form. In contrast, the combination of HPW with NbOPO_4_ or Nb_2_O_5_, regardless of the catalyst form (powder or pellet) or catalytic system (hybrid or impregnated), resulted in the highest HMF concentrations and yields. This superior performance is probably associated with the combination of Brønsted acidity from HPW and Lewis acidity from NbOPO_4_ and Nb_2_O_5_ as both types of acidity are required for the efficient conversion of cellulose into HMF.

**Figure 9:**
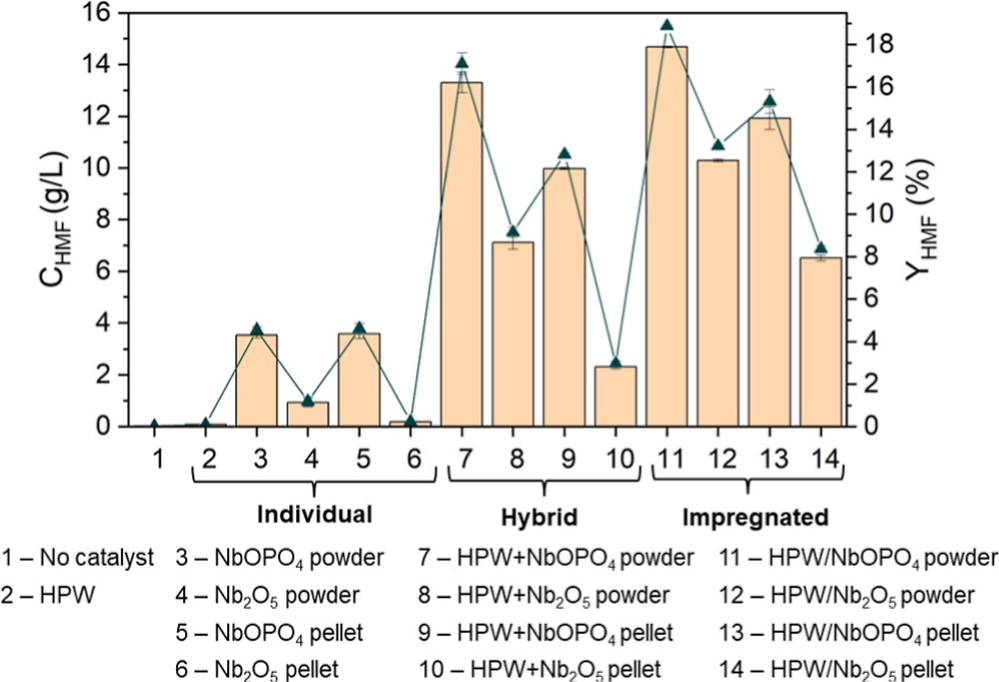
HMF concentration (*C*_HMF_, bars) and yield (*Y*_HMF_, line) from microcrystalline cellulose using different catalytic systems. All reactions were conducted using 10% (w/v) microcrystalline cellulose, an acetone-to-water ratio of 3:1 (v/v), a reaction medium of 100 mL, stirring at 500 rpm, at 200 °C for 10 min. Data represent mean values of duplicate experiments; error bars indicate standard deviations.

Regarding the form of the heterogeneous catalyst, the use of pellets combined with HPW resulted in a lower HMF concentration and yield in hybrid and impregnated systems. This is probably due to the lower surface area of the pellet form and the greater difficulty in achieving effective dispersion in reactions involving pellet-shaped catalysts. This limitation could potentially be mitigated by extending the reaction time or increasing the catalyst loading. Additionally, NbOPO_4_ consistently delivered higher HMF concentrations and yields than Nb_2_O_5_ under all catalytic strategies and catalyst forms evaluated. The best performance was obtained with the impregnated system combining HPW and NbOPO_4_ in the powder form, reaching an HMF concentration of 14.68 g/L and a yield of 18.88% under the studied conditions.

### 3.3 Catalyst Selection for HMF Production from Glucose and Microcrystalline Cellulose

Table 5 summarizes the results of HMF production from glucose and microcrystalline cellulose by using the selected catalysts. NbOPO_4_ powder was selected as the most effective catalyst for glucose conversion, providing the highest HMF concentration and yield. In contrast, NbOPO_4_ powder impregnated with HPW (HPW/NbOPO_4_ powder) exhibited superior performance for microcrystalline cellulose and was therefore selected as the optimal catalyst for its conversion. The selection of distinct catalytic systems is attributed to the structural characteristics of the substrates. The Brønsted acidity introduced by HPW is essential for the conversion of polymeric carbohydrates (e.g., microcrystalline cellulose), whereas it is not required for the dehydration of monomeric sugars, such as glucose.

**Table 5:** HMF Production from Glucose and Microcrystalline Cellulose Using the Selected Catalysts

Substrate	Catalyst	*C*_HMF_ (g/L)	*Y*_HMF_ (%)
Glucose^a^	NbOPO_4_ powder	13.46	38.46
Microcrystalline cellulose^b^	HPW/NbOPO_4_ powder	14.68	18.88

a Results obtained from kinetic study. Reaction condition: 50 g/L of glucose, 3.5% (w/v) catalyst, acetone-to-water ratio of 1:1 (v/v), reaction medium of 100 mL, 500 rpm, 160 °C, and 30 min.

b Reaction condition: 10% (w/v) microcrystalline cellulose, 5% (w/v) catalyst, acetone-to-water ratio of 3:1 (v/v), reaction medium of 100 mL, 500 rpm, 200 °C, and 10 min.

### 3.4 Characterization of the Selected Catalysts

The NbOPO_4_ and HPW/NbOPO_4_ powder catalysts, selected based on their performance in HMF production from glucose and microcrystalline cellulose, respectively, were characterized by X-ray diffraction (XRD), N_2_ adsorption–desorption analysis, and attenuated total reflectance Fourier-transform infrared (ATR-FTIR) spectroscopy with pyridine adsorption.

The XRD patterns of HPW, uncalcined NbOPO_4_, and calcined NbOPO_4_ and HPW/NbOPO_4_ powders (250 °C) are presented in Figure 10. Both NbOPO_4_ samples exhibit only two broad reflections in the 2θ ranges of 15–40° and 40–70°, with no sharp diffraction peaks, indicating their predominantly amorphous nature. This amorphous structure is often associated with a higher surface area and acidity, properties that are advantageous for acid-catalyzed reactions.^36^

**Figure 10:**
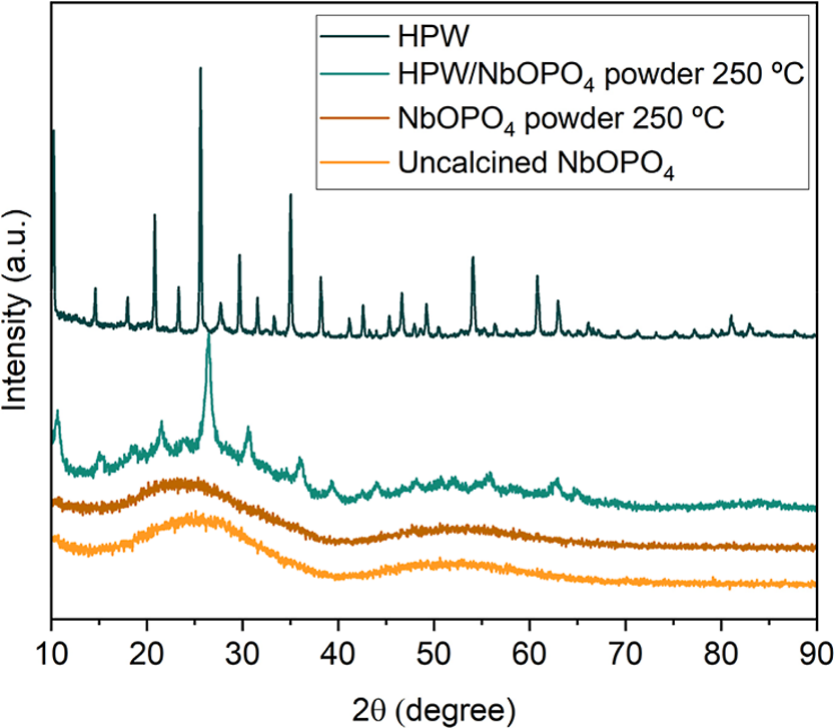
X-ray diffractograms of HPW, uncalcined NbOPO_4_, and NbOPO_4_ and HPW/NbOPO_4_ powders calcined at 250 °C.

Liu et al.,^45^ Junior et al.,^46^ and Bassan et al.^47^ also reported XRD patterns for uncalcined NbOPO_4_ and for samples calcined at higher temperatures (300 and 500 °C). Under these conditions, the authors similarly observed broad patterns typical of amorphous materials, suggesting the morphological stability of NbOPO_4_ even at elevated temperatures. In fact, according to Nowak and Ziolek,^36^ niobium phosphate remains amorphous up to a calcination temperature of 800 °C, above which it transitions to a crystalline phase.

For the HPW/NbOPO_4_ powder catalyst, the diffractogram exhibits a general pattern similar to that of pure NbOPO_4_ powder, but additional reflections corresponding to HPW are also observed, which are characteristic of the Keggin structure.^48^ According to the literature, the presence of such peaks indicates low dispersion of the active phase on the support, probably due to the formation of aggregates in the material.^42^

The textural properties of the previously selected catalysts were also investigated by N_2_ physisorption analysis. The corresponding isotherms are presented in Figure 11, while the specific surface areas, pore volumes, and pore diameters are summarized in Table 6. As shown in Figure 11, both catalysts exhibit type IV isotherms with H1-type hysteresis loops, according to IUPAC classification, indicating the presence of porous solids with a mesoporous structure.^49^ This textural property is particularly relevant for solid catalysts as it facilitates greater accessibility to active sites. A similar isotherm profile was reported by Junior et al.,^46^ who also studied niobium phosphate supplied by CBMM.

**Figure 11:**
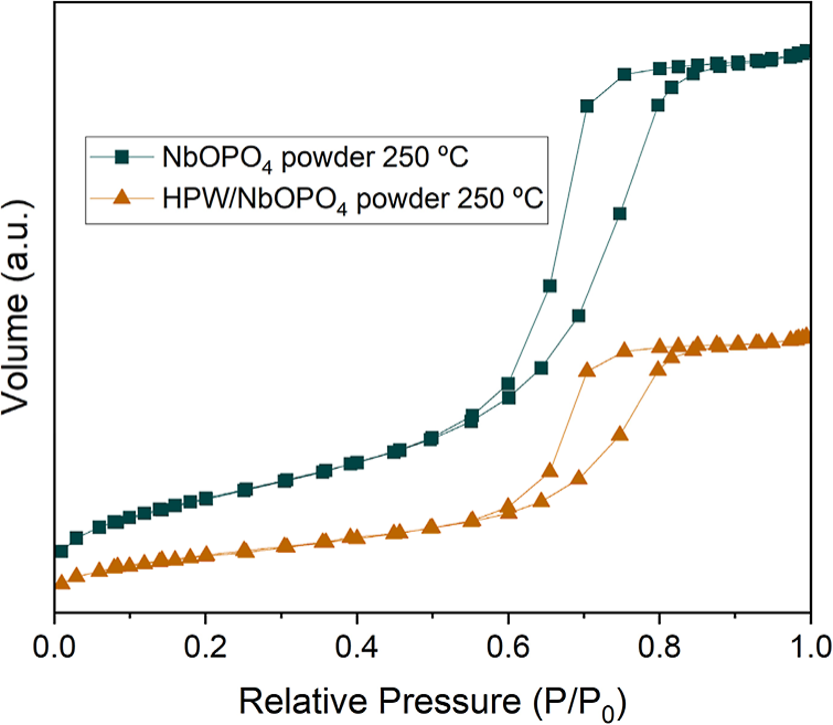
N_2_ adsorption–desorption isotherms of the NbOPO_4_ and HPW/NbOPO_4_ powder catalysts calcined at 250 °C.

**Table 6:** Surface Area, Pore Volume, and Average Pore Diameter of the NbOPO_4_ and HPW/NbOPO_4_ Powder Catalysts Calcined at 250 °C

Catalysts	Surface area (m^2^/g)	Total pore volume (cm^3^/g)	Average pore diameter (nm)
NbOPO_4_ powder 250 °C	162.92	0.32	7.88
HPW/NbOPO_4_ powder 250 °C	87.72	0.16	7

As shown in Table 6, the NbOPO_4_ powder catalyst, calcined at 250 °C, exhibited the highest surface area (162.92 m^2^/g), approximately 86% higher than that of the HPW/NbOPO_4_ powder catalyst (87.72 m^2^/g). Regarding pore volume, NbOPO_4_ showed approximately twice the value observed for the HPW/NbOPO_4_. In terms of pore diameter, the NbOPO_4_ and HPW/NbOPO_4_ powders presented similar average values (7.88 and 7.00 nm, respectively). Junior et al.,^46^ who also used NbOPO_4_ provided by CBMM as a catalyst, reported similar results for surface area (114 m^2^/g), pore volume (0.23 cm^3^/g), and pore diameter (4–10 nm). In contrast, Liu et al.,^45^ who synthesized NbOPO_4_ using four different methods, reported lower values compared to those obtained in the present study, with surface areas ranging from 12.64 to 54.45 m^2^/g, pore volumes from 0.05 to 0.16 cm^3^/g, and average pore diameters between 0.71 and 1.89 nm. These findings emphasize the substantial effect of the catalyst preparation method on its textural properties.

The lower surface area and total pore volume observed for the HPW/NbOPO_4_ catalyst compared to the NbOPO_4_ powder is probably associated with the presence of the HPW Keggin structure within the catalyst pores. At the same time, the preservation of the average pore diameter suggests that the acidic HPW solution did not cause corrosion of the catalyst pores. Similar results were reported by Shen et al.,^42^ who investigated the impregnation of various HPW loadings onto SBA-15. The authors also observed a decrease in surface area and pore volume for the HPW-impregnated catalyst and noted that an increase in HPW content in SBA-15 led to a greater reduction in these parameters, as a larger amount of the HPW Keggin structure occupied the catalyst pores.

The selected catalysts were also characterized in terms of the nature of their acidic sites (Lewis and Brønsted), with the results shown in Figure 12. All catalysts exhibited peaks at 1540 cm^–1^ and 1490 cm^–1^, indicating the presence of Brønsted acidic sites and a combination of Lewis and Brønsted sites, respectively. However, only NbOPO_4_ showed a peak at 1450 cm^–1^, which is typically associated with Lewis acidic sites. These results suggest that the presence of HPW can modify the acidic characteristics of NbOPO_4_. Indeed, HPW is known for its strong Brønsted acidity, and its incorporation into the catalyst may have suppressed the band at 1450 cm^–1^ that was originally observed for pure NbOPO_4_.

**Figure 12:**
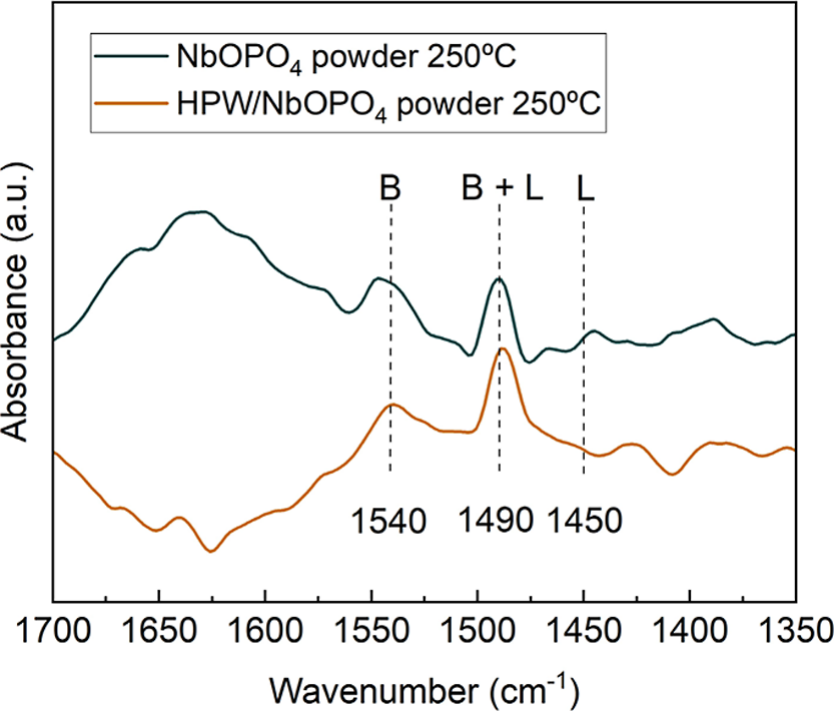
ATR-FTIR spectra of pyridine-adsorbed NbOPO_4_ and HPW/NbOPO_4_ powder catalysts calcined at 250 °C.

Shen et al.,^42^ in a study involving HPW impregnated onto SBA-15 at varying concentrations, similarly did not observe a significant band at 1450 cm^–1^. However, they reported that increasing the HPW content led to an intensified band at 1540 cm^–1^, indicating enhanced Brønsted acidity. Similar results were reported by Kumari et al.^50^ In that study, NbOPO_4_ was impregnated with different HPW loadings, and a higher HPW content led to a greater concentration of Brønsted acidic sites, attributed to the presence of protons within the HPW structure.

These ATR-FTIR results are consistent with the catalytic trends discussed previously and help rationalize the superior performance observed in microcrystalline cellulose conversion for systems containing HPW. In particular, the presence of Brønsted acid sites associated with these catalysts is in line with the known requirements of cellulose hydrolysis, a reaction that typically requires acidic environments due to the polymeric and crystalline nature of the substrate. In this context, the modification of the acidic properties of NbOPO_4_ upon HPW incorporation provides a qualitative basis to discuss the enhanced catalytic performance observed for impregnated catalysts in microcrystalline cellulose conversion. In contrast, the intrinsic acidity of NbOPO_4_, as evidenced by the presence of both Lewis and Brønsted acid sites, appears to be sufficient to promote glucose conversion under the investigated conditions.

### 3.5 Comparative Catalytic Performance in HMF Production from Glucose and Microcrystalline Cellulose

A comparative analysis with recent literature data, presented in Table 7, was performed to further evaluate the catalytic performance of NbOPO_4_ and HPW/NbOPO_4_ in the production of HMF from glucose and microcrystalline cellulose, respectively. To ensure consistency, only studies employing heterogeneous catalysis in batch reactors were considered. Reported HMF yields from glucose typically range from 32% to 90.5%, whereas those from microcrystalline cellulose vary between 13% and 78.9%. In the present study, HMF yields were 38.5% for glucose and 18.9% for cellulose, which fall within the lower range of reported literature values. However, the yield alone does not constitute a comprehensive metric for evaluating catalytic efficiency, especially when considering potential industrial applications.

**Table 7:** Comparison of the Performance of NbOPO_4_-Based Catalysts Investigated in the Present Study with Previously Reported Studies Employing Heterogeneous Catalysis for HMF Production from Glucose and Microcrystalline Cellulose in Batch Reactors

Entry	Substrate	Catalyst	Solvent system	*T* (°C)	*t* (min)	*Y*_HMF_ (%)	*C*_HMF_ ^a^ (g/L)	*Q* ^a^ (g/L·h)	*q* ^a^ (*g*_HMF_/*g* _catalyst_·h)	Ref
1	Glucose (200 mg)	100 mg of Sn–OH/SBA-15	20 mL of THF/saturated NaCl solution volume ratio of 4:1	180	300	70.6	4.94	0.99	0.20	^38^
2	Glucose (0.5 g)	0.5 g of NbOPO – pH7	10 mL of MIBK/water 70:30 (v/v)	140	60	39.3	13.76	13.76	0.28	^51^
3	Glucose (0.1 g)	0.03 g of HfO(PO_4_)_2.0_	5 mL of water/THF 1:4 (v/v) and 0.2 g of NaCl	175	150	90.5	12.67	5.07	0.84	^30^
4	Microcrystalline cellulose (0.1 g)	0.03 g of HfO(PO_4_)_2.0_	5 mL of water/THF 1:4 (v/v) and 0.2 g of NaCl	190	240	69.8	10.86	2.71	0.45	^30^
5	Microcrystalline cellulose (100 mg)	100 mg of AlSiO-20	3.6 g of LiBr and 2.4 g of water	170	20	44.5	14.42	43.26	1.04	^31^
6	Glucose (0.3 g)	0.12 g ofamberlyst-15-Al	0.3 g of ChCl, 0.3 g of water, and 10 mL of MIBK	120	120	64.7	13.19	6.60	0.57	^27^
7	Glucose (0.02 g)	0.02 g of sulfonated carbon/γ-Al_2_O_3_ composites	2.0/0.2 mL of DMSO/H_2_O	160	240	62.3	3.96	0.99	0.11	^15^
8	Microcrystalline cellulose (0.3 g)	0.1 g of 5% Cu–Fe-MMT	18 mL of DMSO and 3 mL of water	170	240	78.9	8.77	2.19	0.11	^39^
9	Glucose (100 mg)	100 mg of γ-AlOOH	2.5 g of DMSO	130	180	61.2	18.85	6.28	0.14	^8^
10	Microcrystalline cellulose (100 mg)	100 mg of γ-AlOOH	4 g of BmimCl, 2 g of DMSO, and 1 mL of water	160	120	58.4	7.32	3.66	0.23	^8^
11	Glucose (100 mg)	20 mg of (10)Hf-DMSNs(2/3)	4 mL of THF, 1 mL of water, and 200 mg of NaCl	190	180	71	9.94	3.31	0.83	^29^
12	Microcrystalline cellulose (100 mg)	20 mg of (10)Hf-DMSNs(2/3)	4 mL of THF, 1 mL of water, and 200 mg of NaCl	210	240	50	7.78	1.94	0.49	^29^
13	Glucose (1 wt %)	10 wt % of 25% Nb–TaP	3 g of 1.0 wt.% glucose solution and 7 g of MIBK	170	180	72.6	1.30	0.43	0.005	^52^
14	Glucose (880 mg)	30 mg of 20% NbOPO_4_/TiO_2_	5 mL of 1:1 dimethyl carbonate/water	170	240	43	52.98	13.24	2.21	^34^
15	Microcrystalline cellulose (440 mg)	30 mg of 20% NbOPO_4_/TiO_2_	5 mL of 1:1 dimethyl carbonate/water	170	300	13	8.90	1.78	0.30	^34^
16	Glucose (3.33 wt %)	0.2 g of NbP@C-RB15	3 mL of H_2_O (NaCl 33%) and 9 mL of MIBK	150	180	32	1.87	0.62	0.037	^35^
17	Glucose (5 g)	3.5 g of NbOPO_4_	50 mL of acetone and 50 mL of water	160	30	38.5	13.46	26.9	0.77	Present Study
18	Microcrystalline cellulose (10 g)	5 g ofHPW/NbOPO_4_	75 mL of acetone and 25 mL of water	200	10	18.9	14.68	88.1	1.76	Present Study

a Values calculated based on the yields provided by the authors. *Q*—volumetric rate. *q*—specific rate.

In this context, the assessment of volumetric (*Q*, g/L·h) and specific (*q*, *g*_HMF_/*g*_catalyst_·h) reaction rates provides a more practical and representative indicator of catalytic performance. The volumetric rates achieved in this study were 2 to 89 times higher than previously reported for glucose, and 2 to 49 times higher for microcrystalline cellulose, demonstrating the superior productivity of the catalytic systems under the investigated conditions, even in comparison with other niobium-based catalytic systems reported in the literature.^34,35,51,52^ Regarding the specific reaction rate, the performance for glucose was comparable to that reported by Shi et al.^29^ and Cao et al.,^30^ and up to seven times higher than those found in other studies included in Table 7. An exception is the study by Kadam et al.,^34^ which reported a specific reaction rate approximately 3-fold (2.87 times) higher than that obtained in the present work. However, the volumetric reaction rate achieved in the present study was about 2-fold higher, indicating superior overall productivity under the investigated conditions. For microcrystalline cellulose, the specific rates were 1.7 to 15 times greater than previously reported values, reinforcing the efficiency of the proposed system even in the presence of a polymeric substrate.

In terms of solvent selection, the literature often reports the use of DMSO and THF,^8,15,29,30,38,39^ both classified as “problematic” according to the CHEM21 solvent guide due to safety, health, and environmental concerns.^40^ In contrast, the present work employed acetone–water mixtures, with acetone contents of 50% or 75% (v/v), depending on the type of substrate (glucose or microcrystalline cellulose). Acetone is a nontoxic, readily biodegradable solvent and is classified as “recommended” by the same guide, representing a safer and more environmentally sustainable alternative. In addition, given its low boiling point (56 °C), acetone could be recovered by distillation and reused in subsequent cycles. This strategy not only aligns with circular economy principles but also could increase the effective HMF concentration in the aqueous phase to 26.9 g/L for glucose and 58.7 g/L for cellulose after solvent removal. These values approach the minimum commercial benchmark of ∼25 wt.% aqueous HMF solutions, as reported by AVA Biochem, the global leader in industrial HMF production.^53^ To reach such levels, additional concentration steps of approximately 10-fold (glucose) and 5-fold (cellulose) would still be required. In this context, acetone removal by distillation may represent a first practical step for solvent recovery and HMF enrichment in the aqueous phase, while a subsequent adsorption step may be considered a promising integrated strategy for further purification, given its operational simplicity, safety, energy efficiency, and environmental compatibility.^54^ Indeed, integrated separation approaches combining distillation and adsorption have been reported to enhance HMF purification and overall process efficiency.^55^ Overall, this highlights the importance of efficient HMF recovery and downstream process optimization toward industrial implementation.

Another important consideration is the use of chloride salts in several studies presented in Table 7, which may hinder scale-up due to their corrosive effects on stainless steel and other reactor materials. In contrast, the systems investigated here operate under noncorrosive conditions, thereby enhancing their suitability for industrial implementation.

Altogether, these findings underscore the potential of NbOPO_4_ and HPW/NbOPO_4_ as promising and sustainable catalytic systems for HMF production, with NbOPO_4_ showing superior performance for the monomeric substrate (glucose) and HPW/NbOPO_4_ being more effective for the polymeric substrate (microcrystalline cellulose). This potential is further supported by the high reaction rates achieved under the investigated conditions, which contribute to improved process performance. In addition, techno-economic analyses of HMF production using niobium phosphate-based catalysts suggest that the contribution of catalyst cost to the overall operating cost may be minor under certain process conditions,^56^ although this is highly dependent on process configuration and operating parameters. The combination of high reaction rates, environmentally friendly and readily recoverable solvents, and noncorrosive conditions highlights their suitability for future biorefinery applications, enabling HMF production directly from lignocellulosic biomass or its hydrolysates. Nevertheless, efficient HMF recovery and concentration remain critical challenges, and further efforts in this direction will be essential to ensure the practical implementation of the proposed methodology on an industrial scale.

## 4 Conclusion

This study demonstrates that the catalytic efficiency in HMF production is influenced by both the structural complexity of the carbohydrate substrate and the composition and physical form of the catalyst. NbOPO_4_ in the powder form enabled a high performance for glucose conversion, while the HPW/NbOPO_4_ combination was essential for the effective conversion of microcrystalline cellulose. This behavior reflects the complementary role of Lewis and Brønsted acidity in promoting glucose dehydration and cellulose hydrolysis. These systems achieved high volumetric (26.9 and 88.1 g/L·h) and specific reaction rates (0.77 and 1.76 *g*_HMF_/*g*_catalyst_·h) for glucose and cellulose conversion, respectively, operating in acetone–water mixtures as a green and noncorrosive solvent system under short reaction times. Additionally, the catalyst’s physical form significantly affected its recyclability, with the powder maintaining activity over seven consecutive glucose conversion cycles without regeneration. While further advances in downstream processing, particularly in solvent recovery and selective HMF separation, will be necessary to reach commercial concentration benchmarks (∼25 wt % aqueous solutions, as reported by AVA Biochem), the catalytic performance demonstrated here contributes to the development of efficient, reusable, and environmentally friendly catalytic systems for carbohydrate valorization. These findings provide a solid foundation for future research on the selective conversion of lignocellulosic biomass within integrated biorefinery systems.
